# Antibacterial Activity of As-Annealed TiO_2_ Nanotubes Doped with Ag Nanoparticles against Periodontal Pathogens

**DOI:** 10.1155/2014/829496

**Published:** 2014-08-18

**Authors:** Sinem Yeniyol, Zhiming He, Behiye Yüksel, Robert Joseph Boylan, Mustafa Ürgen, Tayfun Özdemir, John Lawrence Ricci

**Affiliations:** ^1^Department of Oral Implantology, Faculty of Dentistry, Istanbul University, 34093 Istanbul, Turkey; ^2^Department of Basic Science and Craniofacial Biology, New York University College of Dentistry, New York, NY 10010, USA; ^3^Department of Mechanical Engineering, Istanbul Aydın University, 34668 Istanbul, Turkey; ^4^Department of Metallurgical and Materials Engineering, Faculty of Chemical and Metallurgical Engineering, Istanbul Technical University, 34469 Istanbul, Turkey; ^5^Department of Biomaterials and Biomimetics, New York University College of Dentistry, New York, NY 10010, USA

## Abstract

It is important to develop functional transmucosal implant surfaces that reduce the number of initially adhering bacteria and they need to be modified to improve the anti-bacterial performance. Commercially pure Ti sheets were anodized in an electrolyte containing ethylene glycol, distilled water and ammonium fluoride at room temperature to produce TiO_2_ nanotubes. These structures were then annealed at 450°C to transform them to anatase. As-annealed TiO_2_ nanotubes were then treated in an electrolyte containing 80.7 g/L NiSO_4_ *·*7H_2_O, 41 g/L MgSO_4_ *·*7H_2_O, 45 g/L H_3_BO_3_, and 1.44 g/L Ag_2_SO_4_ at 20°C by the application of 9 V AC voltage for doping them with silver. As-annealed TiO_2_ nanotubes and as-annealed Ag doped TiO_2_ nanotubes were evaluated by SEM, FESEM, and XRD. Antibacterial activity was assessed by determining the adherence of *A. actinomycetemcomitans*, *T. forsythia*, and *C. rectus* to the surface of the nanotubes. Bacterial morphology was examined using an SEM. As-annealed Ag doped TiO_2_ nanotubes revealed intense peak of Ag. Bacterial death against the as-annealed Ag doped TiO_2_ nanotubes were detected against *A. actinomycetemcomitans*, *T. forsythia*, and *C. rectus* indicating antibacterial efficacy.

## 1. Introduction

Dental implants have become a widely applied treatment option in dentistry to replace missing teeth for function and esthetics. However, implant failure and peri-implantitis are problems to be solved to provide long-term stability of the dental implants which depend not only on the integration into the surrounding bone [1], but also on the presence of the protective soft tissue sealing around the implant [2].

The composition, configuration, and density of the proteins in the pellicle derived from the saliva and gingival crevicular fluid are largely dependent on the physical and chemical nature of the underlying surface and thus the properties of the surface influence bacterial adhesion through the pellicle [2]. The phrase “race for the surface” was coined by Gristina in 1987 to describe the competition between bacterial adhesion and tissue integration [3]. If the native host bacteria win the race, tissue cells will not be able to displace these primary colonizers, and biofilm formation will occur developing into peri-implantitis [4, 5]. It is important to develop functional transmucosal implant surfaces that reduce the number of initially adhering bacteria. The first method is to inhibit the initial adhesion of oral bacteria. An ideal transmucosal implant surface exposed to the oral cavity is recommended to be highly polished to resist bacterial colonization and it is expected to allow the formation of an epithelial seal that prevents plaque accumulation leading to peri-implantitis [5, 6]. The second method is to inhibit the colonization of the oral bacteria, which involves surface antibacterial activity [7]. The surface needs modification to optimize the antibacterial properties of the implant. The antibacterial characteristics of implants can be enhanced by mechanical, physical, chemical, and biochemical surface treatments. Electrochemical anodization has been receiving increasing attention as a chemical surface modification method for fabrication of highly ordered nanotubular titanium oxide (TiO_2_) layers for the medical implants as a cost-effective, versatile, and simple technique [8–11]. Anodization leads to an oxidation of metal species that form a solid oxide on the metal surface. Depending on the anodization conditions (potential, nature of the electrolyte, concentration of the electrolyte, temperature, potential sweep rate, pH, and anodizing time) [12, 13], the solid oxide layer can be either compact, or nanotubular [14]. Ordered nanotubular structures of TiO_2_, with a controlled and uniform diameter, length, and wall thickness, can be formed if the dissolution is enhanced by fluoride containing electrolytes and suitable anodization conditions are used [14].

The nanotubular TiO_2_ surface layers play an important role in the improvement of osseointegration through the enhancement of bone cell adhesion, differentiation, ALP activity, bone matrix deposition, apatite deposition rates [15], and hemocompatibility of Ti and Ti-based materials [16–18]. There are also studies validating TiO_2_ nanotubes as promising bioactive coatings with predictable drug release characteristics for local drug delivery systems [19], killing of cancer cells [20], and bacteria cells [21] for anticancer and antibacterial treatments. With the increase of microorganisms resistant to multiple antibiotics, there is a need for alternative antibacterial agents [22]. Silver with its nontoxicity to human cells [23] is widely used as an antibacterial coating to avoid initial adhesion of bacteria onto the implant surface [23, 24]. It is difficult for bacteria to develop resistance against this element. It is effective at low concentrations and relatively large reservoir provided by the nanotubes can give rise to long-term antibacterial effects [25]. With this concept, antibacterial studies have focused on fabrication of TiO_2_ nanotubes serving as carriers for Ag as an antibacterial agent. TiO_2_ nanotubes were loaded with Ag by ultrasonication [26], soaking in AgNO_3_ solutions [25], electrodeposition [27], and sputter deposition techniques [22] to generate surfaces showing adherent Ag nanoparticles uniformly distributed on the TiO_2_ nanotube walls. In our study, electrochemical anodization technique, which is easy and cost effective, was used to fill in the TiO_2_ nanotubes with Ag instead of generating distributed Ag particles on these nanotubes.

Periodontopathogen bacteria can attach intraoral components of implants that are exposed to saliva, plaque, and crevicular fluid and increase the risk for peri-implantitis infections. Therefore, the aim of this study was to indicate the possible clinical benefit of as-annealed Ag doped TiO_2_ nanotubes in providing antimicrobial properties due to their Ag content against the adhesion of peri-implantitis-associated bacteria* Aggregatibacter actinomycetemcomitans*,* Tannerella forsythia*, and* Campylobacter rectus* for transmucosal components of dental implants.

## 2. Materials and Methods

### 2.1. Preparation of Samples

Commercially pure titanium (cpTi) sheets in squares (10 × 10 × 1 mm, 99.6% purity) were used as substrates for the experiments. These sheets were ultrasonically cleaned in acetone, distilled water, and methanol, respectively. The electrochemical anodization was employed to form a layer of TiO_2_ nanotubes on the cpTi sheets. Anodization voltage was kept at 40 V with a DC power supply for 30 min at room temperature. Electrolyte was ethylene glycol with 0.5 wt% ammonium fluoride (NH_4_F) and 3 vol% distilled water [28]. These sheets were then annealed at 450°C in air for 30 min to convert the amorphous TiO_2_ nanotubes into the anatase phase [29]. These sheets containing as-annealed TiO_2_ nanotubes were named as Group TiO_2_. Group TiO_2_ sheets were cleaned with acetone and rinsed with distilled water after anodic oxidation, and they were immediately doped with Ag at 20°C with a constant voltage of 9 V with a DC power supply for 30 sec. Electrolyte contained 80.7 g/L NiSO_4_ *·*7H_2_O, 41 g/L MgSO_4_ *·*7H_2_O, 45 g/L H_3_BO_3_, and 1.44 g/L Ag_2_SO_4_ [30, 31]. The sheets containing as-annealed TiO_2_ nanotubes served as the cathode electrode and a platinum sheet as the counter electrode. These sheets containing as-annealed Ag doped TiO_2_ nanotubes were named as Group Ag. Untreated cpTi sheets were named as Group Ti. 24-well cell culture plate bottoms were used as the control group named as Control Group at the antibacterial assay.

### 2.2. Characterization Methods

The surface morphologies of the sheets were observed using a scanning electron microscope (SEM) (JSM5410, JEOL, Tokyo, Japan) at a 25 kV acceleration voltage. Field emission scanning electron microscope (FESEM) (JSM-7000F, JEOL, Tokyo, Japan) was used to observe the microstructures of the thin films at 5 and 10 kV acceleration voltages and at various magnifications. The structure of the films and corresponding orientations of Ag-TiO_2_ films were determined by utilizing an X-ray diffractometer (Philips PW 3710, Cu-K*α* radiation). A scan rate of 0.01°/sec was used for cpTi surface and as-annealed TiO_2_ films with a grazing incidence of 0.2°, but this grazing incidence was not sufficient to detect the Ag in the as-annealed TiO_2_ nanotubes doped by Ag. For this reason, the grazing incidence used for the as-annealed TiO_2_ nanotubes doped by Ag was as 0.5°.

### 2.3. Antibacterial Assay

The tests were performed using* A. actinomycetemcomitans* (ATCC 43718; ATCC, Rockville MD, USA),* T. forsythia* (ATCC43037A), and* C. rectus* (ATCC 33238). Bacteria cells were cultured in brain heart infusion (BHI) broth (Thermo Scientific Remel, Lenexa, KS, USA) overnight at 37°C. Based on our pilot studies, the bacteria were grown to mid-log phase and centrifuged and resuspended in trypticase soy broth to optical densities of approximately 0.40 for* A. actinomycetemcomitans*, 0.25 for* T. forsythia*, and 0.10 for* C. rectus* at the wavelength of 600 nm. Sheets of the Groups TiO_2_, Ag, Ti, and Control Group were placed into individual wells of the sterile 24-well culture plates with their modified surfaces placed facing upward and bacteria cells were pipetted onto the samples for the three different bacteria experiments. The culture plates were covered by their lids and incubated at 37°C in an anaerobic environment (Modular Atmosphere Controlled System, DW Scientific, Shipley, Yorkshire, UK) for 18 h. The supernatant fluid from each well was appropriately diluted and plated on TSBN media (personal communication, S.S. Socransky, Forsyth Institute, Cambridge, MA, USA) for* A. actinomycetemcomitans*,* T. forsythia*, and* C. rectus* and incubated anaerobically for 4 days at 37°C and the number of colonies (colony-forming unit: CFU) was counted. TSBN was prepared as follows: solution A—26 g of brain heart infusion agar (Thermo Scientific Remel), 20 g of trypticase soy agar (Thermo Scientific Remel), 10 g of yeast extract (Difco Laboratories, Detroit, MI, USA), and 5 mg of hemin (Sigma-Aldrich, St. Louis, MO, USA) were added to 930 mL of distilled water. Solution A was autoclaved and then placed in a water bath. When a temperature of 52°C was reached, the following solutions were added aseptically: 10 mL of a menadione stock solution (5 mg/100 mL) (Sigma-Aldrich), 1 mL of an N-acetylmuramic acid stock solution (1 g/100 mL) (Sigma-Aldrich), and 50 mL of sheep blood (Thermo Scientific Remel).

The cell densities were chosen at different concentrations based on pilot adhesion experiments for being able to view different colonization concentrations of the selected bacteria cells on the material surfaces by SEM. Sheets at the Groups TiO_2_, Ag, and Ti were subjected to fixation followed by SEM as described next.

### 2.4. Bacterial Morphology

Representative sheets colonized with the selected oral bacteria were prepared for SEM following standard procedures. Sheets were fixed in 2.5% glutaraldehyde for 1 h. After washing three times in the 0.1 M phosphate buffer, bacteria were postfixed with 1% OsO_4_ for 1 h. After sheets were rinsed twice in the 0.1 M phosphate buffer, they were dehydrated through a graded alcohol series (25–100%). Hexamethyldisilazane was applied twice. Sheets were subsequently critical-point dried; sputter coated with gold, and examined using SEM (Philips XL 30, Eindhoven, The Netherlands) at 20 and 25 kV accelerating voltages.

### 2.5. Statistical Analysis

Statistical analysis was done online with VassarStats: Statistical Computation Web Site. Differences in the mean numbers of the microbes (CFUs) harvested from the experimental materials were tested with one-way analysis of variance (ANOVA) and post hoc analyses were performed using the Tukey's studentized range (HSD) test. All results were reported as mean ± standard deviation (SD). Threshold for significance was set for *P* < 0.05.

## 3. Results and Discussion

The goal of this study was to develop an antibacterial surface for the transmucosal components of dental implants less prone to periodontopathogen bacteria colonization. This objective was achieved via surface nanostructural modification by electrochemical anodization and annealing followed by Ag doping. This study showed that as-annealed Ag doped TiO_2_ nanotubes inhibited adhesion of* A. actinomycetemcomitans*,* T. forsythia*, or* C. rectus*.

It is generally accepted that the periodontopathogen bacteria play a crucial role in peri-implantitis through an assembly of putative virulent factors.* A. actinomycetemcomitans* is responsible for the induction of inflammation of the gingivae and destruction of the periodontal ligament and alveolar bone by modulating inflammation, inducing tissue destruction, and inhibiting tissue repair [32]. Epithelial cell invasion by* T. forsythia* is considered to be an important virulence mechanism and it has putative virulent factors such as trypsin-like protease, sialidase, hemagglutinin, components of the bacterial S-layer, and cell surface-associated and secreted protein (BspA) [33].* C. rectus* is a bacterium reaching the deeper parts of the subgingival pockets using the motility of its flagellum that appear to be the major pathogenic factor [34]. To examine the antimicrobial properties of the surfaces,* A. actinomycetemcomitans*,* T. forsythia*, and* C. rectus* were chosen for this study as they are bacterial species associated with periodontal disease considering their individual putative virulent factors.

**Figure 1:**
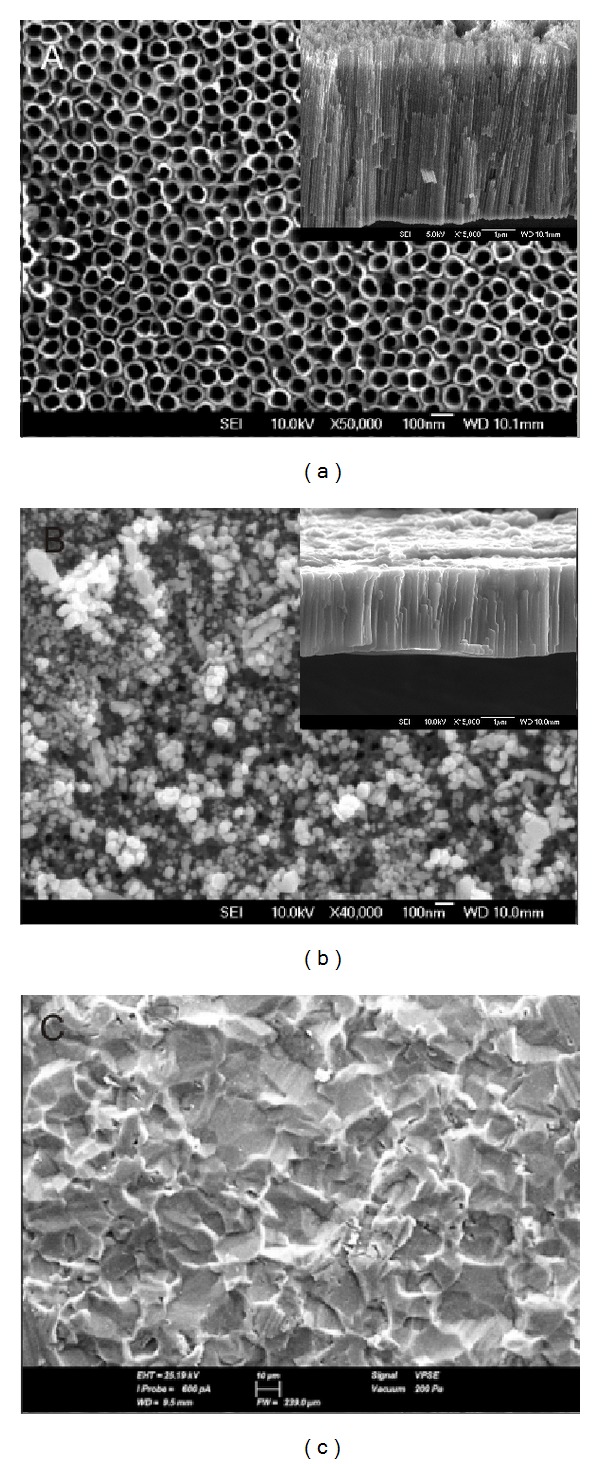
(a) Top-view FESEM micrograph of the surface of Group TiO_2_ displaying as-annealed TiO_2_ nanotubes (bar = 100 nm). Inset: side view of the as-annealed TiO_2_ nanotubes (bar = 1 *μ*m). (b) Top-view FESEM micrograph of the surface of Group Ag displaying as-annealed Ag doped TiO_2_ nanotubes (bar = 100 nm). Inset: side view of the as-annealed Ag doped TiO_2_ nanotubes (bar = 1 *μ*m). (c) Top-view SEM micrograph of the surface of Group Ti displaying cpTi sheet (bar = 10 *μ*m).

**Figure 2:**
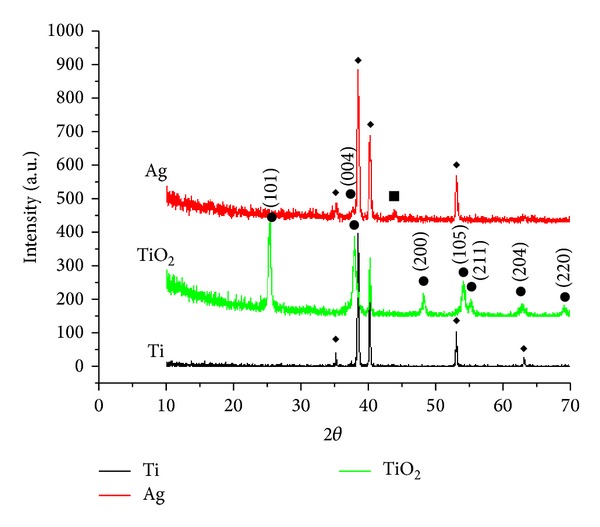
X-ray diffraction patterns of TiO_2_: as-annealed TiO_2_ nanotubes at Group TiO_2_; Ag: as-annealed Ag doped TiO_2_ nanotubes at Group Ag (◆: Ti; ●: anatase; ■: Ag); Ti: cpTi surface at Group Ti.

**Figure 3:**
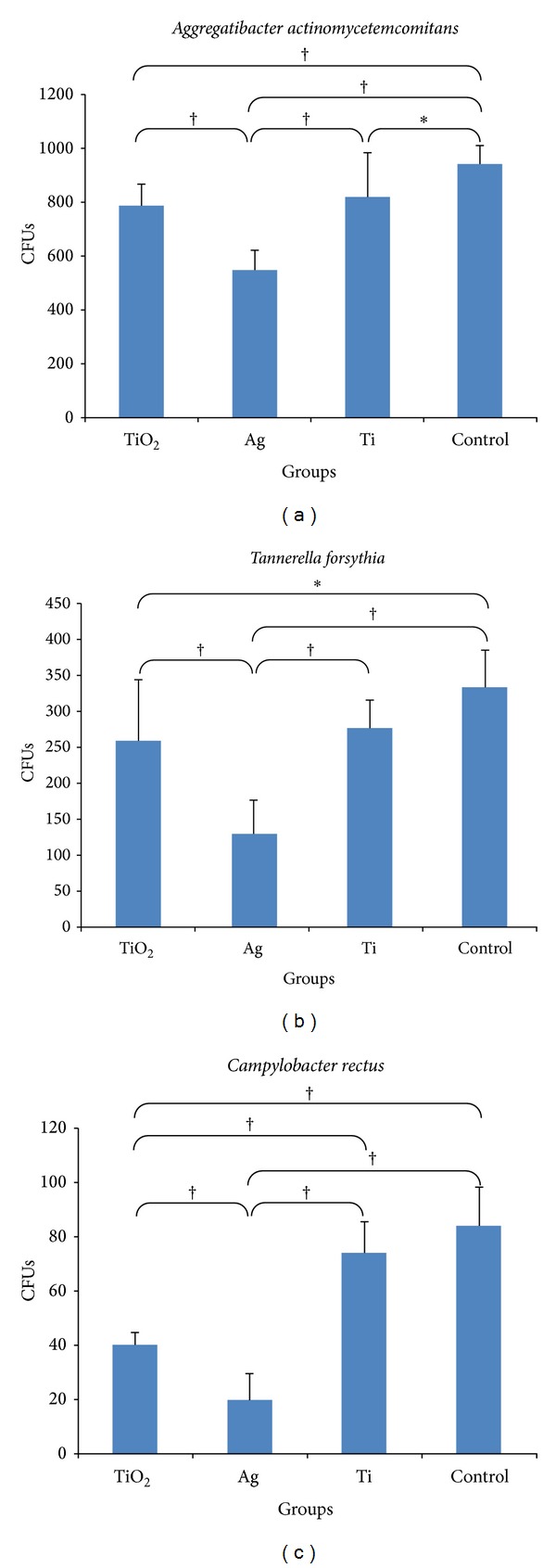
Descriptive analysis of adhesion of (a)* A. actinomycetemcomitans*, (b)* T. forsythia*, and (c)* C. rectus* on all groups tested (Group TiO_2_: as-annealed TiO_2_ nanotubes; Group Ag: as-annealed Ag doped TiO_2_ nanotubes; Group Ti: commercially pure Ti sheet; Control Group: 24-well cell culture plate bottoms). Data are presented as the mean ± SD (standard deviation). Results were analyzed using a one-way ANOVA and post hoc analyses were performed using Tukey's studentized range (HSD) test (*P < 0.05 and ^†^ *P* < 0.01).

In our study, cpTi surfaces were observed to be smooth, with features of grooves, valleys, and peaks at the micron scale before electrochemical anodization (Figure 1(c)). These cpTi surfaces were electrochemically anodized with ethylene glycol, distilled water, and ammonium fluoride to produce highly ordered TiO_2_ nanotube formation on the Ti substrates. Vertically orientated as-annealed TiO_2_ nanotubes, with an inner diameter of 70–100 nm as grown on Ti substrates after electrochemical anodization, were formed in Group TiO_2_ (Figure 1(a)). The side view image (inset of Figure 1(a)) indicated that the as-annealed TiO_2_ nanotubes were straight with uniform pore walls opened at the top with a length of 4.5 *μ*m. Photoresponse of these nanotubes is affected by tube geometry (length, diameter, and tube wall thickness) and structure (anatase, anatase/rutile). The longer the tube, the larger the surface area with higher total light absorption [14]. TiO_2_ is usually used as a photocatalyst in two crystal structures: rutile and anatase. Anatase generally has much higher activity than rutile [35]. As-synthesized nanotubes are amorphous, and postannealing is required to crystallize them into anatase, rutile, or bookite structure [12]. They can be crystallized into anatase at temperatures higher than approximately 280°C in air or a mixture of anatase and rutile at temperatures higher than approximately 450°C [14, 36–39]. In this regard, we aimed to form crystallized TiO_2_ nanotubes by annealing the sheets at 450°C after electrochemical anodization. This is confirmed by the results in the XRD pattern showing several dominant peaks of anatase phase after annealing process. They indicate diffraction peaks at 2*θ* = 25.5°, 38.1°, and 48.3° that are identified to be (1 0 1), (0 0 4), and (2 0 0) crystal faces, respectively. All the anodized TiO_2_ films after the annealing process contained anatase (JCPDS 21-1272) without any evidence for rutile structure (Figure 2). Anodization of cpTi surfaces (Group Ti) clearly diminished the adhesion of* C. rectus* on as-annealed TiO_2_ nanotubes (Group TiO_2_) (*P* < 0.05), whereas no significant differences were found in* A. actinomycetemcomitans* and* T. forsythia* adhesion (*P* > 0.05; all) (Figure 3). UV exposure for photocatalytic activity was not applied in our experiments. It is widely known that TiO_2_ photocatalysts have minimal antibacterial efficacy in visible light [40]. Antibacterial effect against selected periodontopathogen bacteria on the as-annealed TiO_2_ nanotubes can be ascribed to either the visible light exposure or the bacterial sensitivity of* C. rectus* against the crystal structure of anatase.

**Figure 4:**
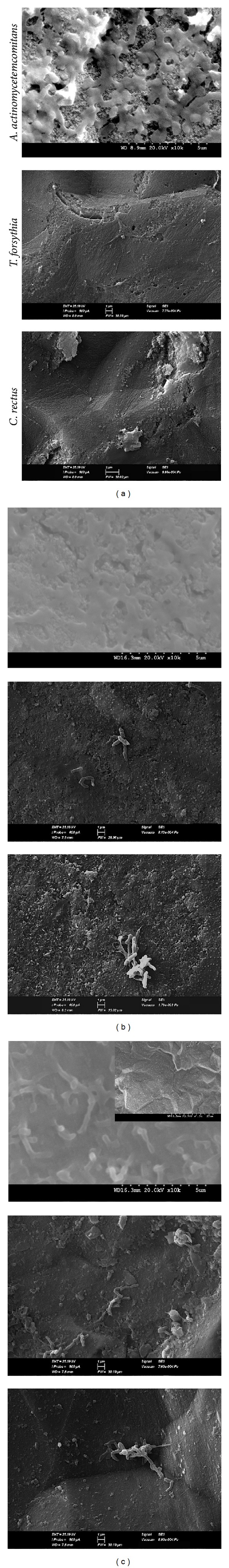
SEM micrographs after adhesion of* A. actinomycetemcomitans*,* T. forsythia*, and* C. rectus* on the surface of (a) Group TiO_2_: as-annealed TiO_2_ nanotubes; (b) Group Ag: as-annealed Ag doped TiO_2_ nanotubes; (c) Group Ti: commercially pure Ti sheet.

The high efficacy of Ag at very low concentrations and the relatively large reservoir provided by the nanotubes offer long-term antibacterial effects. In our study, vertically aligned as-annealed TiO_2_ nanotubes as grown on Ti-surfaces doped with Ag were formed in Group Ag (Figure 1(b)). The side view image (inset of Figure 1(b)) indicated that the as-annealed Ag doped TiO_2_ nanotubes were grown in vertical direction in length up to 6 *μ*m after doping. The mechanism of this reservoir was reported by Zhao et al. [25] as the release of the oxidized Ag^+^ by the slow infiltration of body fluids into the nanotubes leading to antibacterial effect. They considered selecting TiO_2_ nanotubes with a size smaller than 130 nm which should reduce water infiltration and accomplish controlled release of Ag^+^. In accordance with this finding, controlled release of Ag^+^ was expected for the nanotubes in our study with a diameter of 70–100 nm for the antibacterial effect. According to our data, The XRD pattern exhibited diffraction peaks in the pattern corresponding to anatase phase of TiO_2_ and cpTi surface while the small peak at 2*θ* = 44.5 which was allocated to the diffraction of (2 0 0) plane of face centered cubic (FCC) silver marked with Ag (Figure 2) [41]. Anodized surfaces with as-annealed Ag doped TiO_2_ nanotubes (Group Ag) did not enhance* A. actinomycetemcomitans*,* T. forsythia,* and* C. rectus* adhesion on titanium surface compared to the Control Group (*P* < 0.01; all) (Figure 3). This reduction in bacterial activity on the as-annealed Ag doped TiO_2_ nanotubes showed that the use of as-annealed TiO_2_ nanotubes containing Ag was effective in improving the antimicrobial properties of Ti-based materials. In accordance with our findings, on the surfaces of the Group TiO_2_ and Group Ag, bacteria cells showed highly deteriorated morphologies (Figures 4(a) and 4(b)), while bacteria cells at Group Ti showed no morphological change indicating bacterial death (Figure 4(c)). As a consequence of bacterial deterioration, surfaces at Group TiO_2_ and Group Ag were covered with dead biofilms composed of the bacteria remnants.

Antibacterial action of silver is not yet well understood, but it is suggested that Ag nanoparticles can cause bacterial penetration through interaction with sulfur-containing proteins at the bacterial membrane and the phosphorus containing compounds like DNA, finally leading to cell death by attacking the respiratory chain. Ag nanoparticles also release Ag^+^ ions enhancing their bactericidal activity by converting DNA from relaxed state into its condensed form and thereby preventing its replication which would lead to cell death [42]. It is reported that cell proliferation, adhesion, and spreading are improved by the TiO_2_ layer. Therefore, it was suggested that the combination of antibacterial properties (from Ag) and biocompatibility (from TiO_2_) of the TiO_2_/Ag compound coating might be advantageous for medical use [43]. In this regard, titanium surface can be modified by TiO_2_ nanotubes to enhance bone-cell materials interactions, and the nanoporous surface can then be silver coated to further improve antibacterial activity on the surface [44]. According to the investigators, eukaryotic cells show more structural and functional redundancy as bigger targets for attacking silver ions compared to prokaryotic cells acquiring higher silver ion concentrations to achieve comparable toxic effects, relative to the bacteria cells [45]. Though there are limited reports about the antimicrobial effect of the nanostructure of materials, the results of the research by Zheng et al. [46] suggest that not only was Ag-implanted titanium reported to promote osteogenesis with increased cell attachment, viability, and osteogenic gene expression, but also it appeared to show a strong antimicrobial effect against oral microorganisms including* S. mutans*,* P. gingivalis*, and* C. albicans*. Das et al. [44] revealed that Ag-treated TiO_2_ nanotube surface provided antibacterial properties against* P. aeruginosa* without interference to the attachment and proliferation of human osteoblasts. Ewald et al. [47] achieved the establishment of titanium/silver hard coatings via physical vapor deposition with significant antimicrobial potency and absence of cytotoxical effects on osteoblasts and epithelial cells. The antibacterial resistance demonstrated in our study is consistent with the previous studies demonstrating antimicrobial effects of silver ions which make them promising for combating postoperative infection for application in dental implant placement procedures.

## 4. Conclusions

It is of great importance to provide antibacterial activity for maintaining plaque-free surfaces on transmucosal parts exposed to the oral cavity as a future strategy. This study demonstrates that the use of electrochemical anodization and Ag doping provides the required antibacterial surface properties against the selected periodontal pathogens,* A. actinomycetemcomitans*,* T. forsythia*, and* C. rectus*, resulting in reproducible antibacterial coatings on transmucosal parts of dental implants. The findings, however, have to be verified in clinical settings.
